# Supplementary material for: The Association between Fasting Glucose and Sugar Sweetened Beverages Intake Is Greater in Latin Americans with a High Polygenic Risk Score for Type 2 Diabetes Mellitus
Source: Nutrients. 2021 Dec 24;14(1):69. doi: 10.3390/nu14010069 (PMC8746587; doi:10.3390/nu14010069)
Supplement: Supplementary file 1 [file nutrients-14-00069-s001.zip › nutrients-1450768-supplementary.pdf]

Supplementary Material

Supplementary Table S1. SNPs selected from the literature.

SNP	Gene	Chr:Position	PA	RA	OR	P	BF	Cases/Controls	Eth	Bibliography
rs3923113	GRB14	2:165501849	C	A	1.17	3.5E-04	IR	2,499/5,247	L	PMID: 28254843
rs1801282	PPAR-γ	10:70931474	G	C	1.11	2.4E-19	IR	73,337/192,314	T-E	PMID: 28869590
rs11717195	ADCY5	3: 123082398	C	T	1.24	5.1E-06	BCF	2,499/5,247	L	PMID: 28254843
rs4402960	IGF2BP2	3: 185511687	G	T	1.16	8.1E-04	BCF	2,499/5,247	L	PMID: 28254843
rs6878122	ZBED3	5:76427311	A	G	1.17	1.7E-04	BCF	2,499/5,247	L	PMID: 28254843
rs7756992	CDKAL1	6: 20679709	A	G	1.13	2.6E-03	BCF	2,499/5,247	L	PMID: 28254843
rs849135	JAZF1	7: 28196413	A	G	1.19	2.1E-05	BCF	2,499/5,247	L	PMID: 28254843
rs516946	ANK1	8: 41519248	T	C	1.22	3.7E-05	BCF	2,499/5,247	L	PMID: 28254843
rs17791513	TLE4	9:81905590	G	A	1.21	3.0E-08	BCF	26,488/83,964	T-E	PMID:24509480
rs2796441	TLE1	9:84308948	A	G	1.14	1.2E-03	BCF	2,499/5,247	L	PMID:28254843
rs11257655	CDC123	10: 12307894	C	T	1.21	2.0E-05	BCF	2,499/5,247	L	PMID: 28254843
rs7903146	TCF7L2	10:114758349	C	T	1.36	1.3E-11	BCF	2,499/5,247	L	PMID: 28254843
rs10830963	MTNR1B	11: 92708710	C	G	1.28	1.5E-07	BCF	2,499/5,247	L	PMID: 28254843
rs7403531	RASGRP1	15:38822905	C	T	1.21	6.3E-06	BCF	2,499/5,247	L	PMID: 28254843
rs7202877	BCAR1	16: 75247245	G	T	1.06	4.0E-06	BCF	73,337/192,314	T-E	PMID:28869590
rs5945326	DUSP9	X:153642492	G	A	1.20	4.0E-04	BCF	26,676/132,532	Eur	PMID:28566273
rs188827514	CCND2	12: 4291596	G	A	1.64	6.4E-6	BCF	26,676/132,532	Eur	PMID:28566273

SNPs selection for sequencing from OR reported in GWAS studies in Latin and European population. PA: Protection Allele, RA: Risk Allele, OR: Odds Ratio, BF: Biological Function, IR: Insulin resistance, BCF: Beta cell function, Eth: Ethnicity, L: Latinos, Eur: European, T-E: Trans-ethnic.

Suppementary Table S2. Analysis of Variance for log-glycemia.

Variable	Df	SSQ	F	P
Sex	1	0.31244	15.9680	**6.63e-05**
Age (years)	1	0.78047	39.9665	**0.07e-10**
WHC (cm)	1	1.41268	72.3402	**<2.2e-16**
Education (low/Medium/High)	2	0.07975	20.420	0.12999
Socioeconomic (% low/med/high)	1	0.02548	13.045	0.25350
Physical Activity (score)	1	0.02222	11.380	0.28619
Smoking (Never/Current/Former)	1	0.12046	61.686	**0.01307**
Fruit intake ≥1 portions/day	1	0.01100	0.5631	0.45310
Vegetables intake ≥1 portions/day	1	0.04976	25.480	0.11057
Processed meat ≥ 4 portions/week	1	0.00834	0.4272	0.51343
Sugar ≥4 teaspoons/day	1	0.00875	0.4479	0.50337
High risk alcohol consumption	1	0.01816	0.9299	0.33499
GRS-w	2	0.36782	9.3993	**8.58e-05**
Amerindian Ancestry	1	0.04898	2.5034	0.11373
SSB category	3	0.23592	4.0191	**0.00728**
Sex x GRS-w	2	0.01912	0.4897	0.61289
Sex x SSB category	3	0.04538	0.7746	0.50809
GRS-w x Amerindian ancestry	2	0.00205	0.0525	0.94889
Amerindian Ancestry x SSB category	3	0.02810	0.4797	0.69643
GRS-w x SSB category	6	0.30890	26.363	**0.01499**

F tests for each factor were performed by comparing the full model with a reduced model that contained all other variables. Marginal effects of variables participating in an interaction were tested in a model without the interaction. No correction for multiple testing was performed, n=3321 for environmental and lifestyle variables and 2828 for genomic variables.

Supplementary Table S3. Association between selected variables and log-fasting glucose.

**Variable**	**β (SE)**	**IC (95%)**	***P***	**df**	**MS**	***F***	***P***
age	0.002 (0.0003)	0.001 - 0.003	0.0001	1	1.10	50.3	**0.0001**
sex (women)	-0.03 (0.007)	-0.06 - 0.04	0.0001	1	0.03	87.62	**0.0001**
BMI	0.004 (0.0004)	0.004 - 0.005	0.0001	1	1.32	121.05	**0.0001**
WC	0.003(0.0002)	0.002 - 0.003	0.0001	1	2.89	15.55	**0.0001**
Education Medium	0.01 (0.006)	0.001 - 0.024	0.03	2	-	-	-
Education High	0.005 (0.009)	-0.01 - 0.022	0.54	-	0.035	1.90	0.08
Socioeconomic level 1 (Medium)	0.001 (0.006)	-0.01 - 0.01	0.84	2	-	-	-
Socioeconomic level 2 (High)	0.01 (0.009)	-0.01 - 0.01	0.10	2	0.04	1.69	0.18
Physical Activity	-0.002 (0.002)	-0.006 - 0.001	0.18	1	0.04	1.77	0.18
Smoke (current)	-0.01 (0.006)	-0.02 - 0.002	0.10	-	-	-	**-**
Smoke (past)	0.31 (0.007)	0.02 - 0.05	0.0001	2	0.38	17.81	**0.0001**
Fruit (≥portion/day)	0.004 (0.005)	-0.007 - 0.01	0.45	1	0.01	0.55	0.45
Vegetables (≥portion/day)	0.005 (0.006)	-0.006 - 0.02	0.33	1	0.02	0.94	0.33
Processed meat (4 ≥portion/week)	0.003 (0.009)	-0.01 - 0.02	0.72	1	0.002	0.13	0.72
Sugar (4 ≥portion/day)	-0.0004 (006)	-0.01 - 0.01	0.94	1	0.001	0.01	0.93
High alcohol consumption	0.007 (0.007)	-0.007 - 0.02	0.35	1	0.018	0.86	0.35
AMR (%)	0.03 (0.03)	-0.03 - 0.10	0.33	1	0.02	0.94	0.33
w-GSR Tertil 2	0.004 (0.004)	-0.009 - 0.02	0.53	2	0.13	6.03	**0.0024**
Tertil 3	0.03 (0.007)	0.009 - 0.03	0.0001				
SSB Category 2	0.006 (0.007)	-0.008 - 0.02	0.38	3	0.15	6.94	**0.0001**
Category 3	0.01 (0.008)	0.002 - 0.03	0.02	-	-	-	-
Category 4	0.04 (0.009)	0.02 - 0.06	0.0001	-	-	-	**0.0001**
Tryglicerides (mg/dl) (log)	0.007 (0.005)	0.06 - 0.07	0.0001	1	1.10	3.70	**0.0001**
HDL-c (mg/dl)	-0.0009 (0.002)	-0.001 - -0.0005	0.0001	1	0.35	16.59	**0.0001**

Results of linear regressions models are shown as **β**, Standard Error (SE) and 95% CI (Confidence Interval). T: tertil, Cat=SSB category, WC: waist circumference (cm), AMR: Amerindian ancestry, high alcohol consumptiom: male ≥30 & women ≥20 (portions/week), df= degree of freedom, MS=No correction for multiple testing was performed. P-values in bold mean significance ($p < 0.05$). No correction for multiple testing was performed, n=3321.

Supplementary Table S4. General characteristics of all participants by category of sugar-sweetened beverage intake.

Variable	servings/day				*P*
	0=0	>0&<1	1≤&<2	≥2	
N (Total 2828)	686	1064	710	368	
Sex (% Women)	81.6 (560)	65.3 (695)	62.0 (440)	44.0 (162)	**0.0001**
Age (years)	55.6 (9.8)	53.3 (9.4)	52.5 (9.2)	50.5 (8.5)	**0.03**
Education (level) (%) (n)					
Low	46.5 (303)	50.8 (521)	49.3 (338)	47.3 (168)	0.73
Medium	41.3 (269)	37.9 (389)	38.9 (267)	39.7 (141)	
High	12.2 (79)	11.3 (116)	11.8 (81)	13.0 (46)	
Socioeconomic Status (%) (n)					
Low	24.0 (164)	22.7 (241)	26.3 (187)	25.6 (94)	0.40
Medium	57.1 (390)	60.8 (644)	58.0 (411)	57.8 (212)	
High	18.9 (129)	16.5 (175)	15.7 (111)	16.6 (61)	
Physical activity after work (%) (n)					
Yes	19.4 (120)	20.0 (201)	19.7 (132)	20.4 (72)	0.90
Smoke (%) (n)					
Never	49.7 (336)	44.8 (467)	38.8 (271)	33.0 (120)	**0.001**
Current	26.9 (182	31.4 (328)	34.9 (244)	46.4 (169)	
Past	23.4 (158)	23.8 (248)	26.3 (184)	20.6 (75)	
Ancestry (%) (n)					
Amerindian	0.34 (0.08)	0.35 (0.07)	0.36 (0.07)	0.35 (0.07)	**0.003**
European	0.62 (0.09)	0.61 (0.08)	0.59 (0.08)	0.60 (0.08)	**0.004**
African	0.04 (0.04)	0.04 (0.03)	0.05 (0.03)	0.05 (0.03)	0.14
Alcohol consumption (%) (n) (Men ≥30 mL/week & Women ≥20 mL/week)					
High risk	12.6 (85)	14.0 (145)	16.1 (113)	23.7 (81)	**0.0001**
Fruit intake (%) (n)					
<1portions/day	38.8 (266)	55.7 (602)	53.9 (383)	60.6 (223)	**0.0001**
Vegetables intake (%) (n)					
< 1portions/day	23.6 (162)	34.7 (365)	30.4 (216)	40.2 (220)	**0.0001**
Processed meat (%) (n)					
≥ 4 portions/week	4.2 (29)	8.8 (91)	13.7 (95)	17.4 (61)	**0.0001**
Sugar intake (%) (n)					
≥ 4 teaspoons/day	22.6 (154)	39.2 (403)	47.1 (324)	52.2 (187)	**0.0001**
BMI (kg/m2)	29.0 (5.1)	29.3 (4.6)	29.5 (4.7)	29.6 (4.7)	**0.004**
BMI (category) (%) (n)					
Normal	18.8 (129)	17.1 (182)	14.6 (104)	15.5 (57)	**0.14**
Overweight	45.8 (314)	43.6 (464)	44.8 (318)	41.8 (154)	
Obese	35.4 (243)	39.3 (418)	40.6 (288)	42.7 (157)	
Waist Circumference (cm)	96 (11.4)	98.2 (10.8)	98.9 (10.1)	99.9 (10.4)	**0.0001**
Fasting blood glucose (mg/dl)	94.5 (18.2)	94.8 (14.0)	96.2 (17.6)	99.8 (31.9)	**0.0001**
Fasting blood glucose (log)	4.53 (0.14)	4.54 (0.12)	4.55 (0.15)	4.57 (0.20)	**0.0001**
Fasting blood glucose (category) (%) (n)					
≥ 100 mg/dl	19.7 (135)	20.2 (215)	24.2 (172)	26.1 (96)	**0.02**
Triglicerydes (mg/dl)	151.5 (90.0)	163.6 (121.0)	164.8 (116.7)	177.9 (176.4)	**0.0001**

HDL-c (mg/dl)	47.80 (11.5)	45.14 (10.5)	46.15(11.3)	43.15 (10.7)	**0.04**

Data are expressed as mean and Standard Deviation (SD) or percentage (%). Chi-square tests for categorical variables and ANOVA or Kruskal-Wallis tests for continuous variables. P-values in bold mean significance ($p < 0.05$). No correction for multiple testing was performed.

Supplementary Table S5. Association between SSB intake and fasting glucose (log) in whole sample, men and women.

	Servings/day	BMI/WC non adjusted			BMI Adjusted		
		**β (SE)**	**IC (95%)**	**P**	**β (SE)**	**IC (95%)**	**P**
All	>0 & <1	0.007 (0.008)	-0.008-0.02	0.32	0.005 (0.008)	-0.01-0.02	0.48
	1≤&<2	0.02 (0.009)	-0.0004-0.03	0.055	0.013 (0.008)	-0.004-0.03	0.13
	≥2	0.04 (0.01)	0.02-0.06	**0.0001**	0.04 (0.01)	0.01-0.06	**0.001**
Men	>0 & <1	-0.01 (0.02)	-0.05-0.02	0.46	-0.01 (0.02	-0.05-0.02	0.49
	1≤&<2	0.007 (0.02)	-0.03-0.05	0.72	0.008 (0.02)	-0.03-0.05	0.68
	≥2	0.03 (0.02)	-0.01-0.07	0.19	0.03 (0.02)	-0.01-0.07	0.21
Women	>0 & <1	0.01 (0.008)	-0.002-0.03	0.08	0.01 (0.008)	-0.004-0.03	0.15
	1≤&<2	0.02 (0.009)	-0.001-0.04	0.33	0.01 (0.009)	-0.007-0.03	0.20
	≥2	0.05 (0.01)	0.02-0.07	**0.001**	0.04 (0.01)	0.01-0.06	**0.003**

Linear regression results expressed as β (SE) adjusted for age, sex, BMI, WC, educational level, socio-economic level, physical activity, % Amerindian ancestry, smoking, consumption of: fruit, vegetables, processed meat, sugar and alcohol in the whole sample and stratifying by sex. No correction for multiple testing was performed. P-values in bold mean significance (< 0.05). No correction for multiple testing was performed. BMI= Body Mass Index, WC= waist circunference, n=3241. P values in bold are significant ($p < 0.05$).

Supplementary Table S6. Association between SNPs-T2D and fasting glucose (log) in all participants.

SNP	Gene	β (BMI-adjusted)	CI 95%	P	β (non BMI-adjusted)	CI 95%	P
rs17791513	TLE4	0.005 (0.007)	-0.009 - 0.020	0.47	0.005 (0.007)	-0.01 - 0.01	0.47
rs2796441	TLE1	0.001 (0.005)	-0.009 - 0.012	0.80	0.001 (0.005)	-0.009 - 0.012	0.80
rs5945326	DUSP9	0.001 (0.005)	-0.008 - 0.011	0.81	0.001 (0.005)	-0.008 - 0.011	0.81
rs3923113	GRB14	0.012 (0.006)	-0.001 - 0.025	0.07	-0.001 (0.008)	-0.001 - 0.025	0.07
rs516946	ANK1	0.02 (0.007)	0.012 - 0.041	**0.0004**	0.020 (0.007)	0.011 - 0.041	**0.0004**
rs1801282	PPAR-γ	0.006 (0.009)	-0.012 - 0.024	0.66	0.004 (0.009)	0.004 - 0.667	0.66
rs6878122	ZBED3	0.006 (0.005)	-0.005 - 0.017	0.27	0.006 (0.006)	-0.005 - 0.018	0.27
rs7756992	CDKAL1	-0.007 (0.005)	-0.018 - 0.004	0.18	-0.007 (0.005)	-0.019 - 0.003	0.18
rs849135	JAZF1	-0.002 (0.005)	-0.013 - 0.009	0.50	-0.003 (0.005)	-0.015 - 0.007	0.50
rs10830963	MTNR1B	0.006 (0.007)	-0.013 - 0.009	0.45	0.005 (0.007)	-0.015 - 0.007	0.45
rs11717195	ADCY5	0.009 (0.23)	-0.003 - 0.021	0.23	0.007 (0.006)	-0.005 - 0.020	0.23
rs4402960	IGF2BP2	0.022 (0.006)	0.010 - 0.034	**0.0007**	0.021 (0.006)	0.008 - 0.033	**0.0007**
rs11257655	CDC123	0.001 (0.006)	-0.012 - 0.014	0.86	0.001 (0.006)	-0.012 - 0.014	0.23
rs7903146	TCF7L2	0.024 (0.006)	0.011 - 0.037	**0.0009**	0.021 (0.006)	0.009 - 0.035	**0.0009**
rs7403531	RASGRP1	0.007 (0.006)	-0.005 - 0.019	0.23	0.007 (0.006)	-0.005 - 0.020	0.23
rs7202877	BCAR1	0.0001 (0.008)	-0.016 - 0.016	0.88	-0.001 (0.008)	-0.018 - 0.015	0.88

Results are shown with β, 95% CI (Confidence Interval). All models were adjusted for age, sex and Amerindian ancestry (n=3378). Body Mass Index (BMI) was included and excluded for models. P values in bold are significant after Bonferroni correction (p<0.0031), n=3866.

Supplementary Table S7. Interaction effects between categorical SSB intake and additive effects from D2M SNPs on fasting glucose.

rsID	Gene	SSB Category 2		SSB Category 3		SSB Category 4		
		**β (SE)**	**P_t**	**β (SE)**	**P_t**	**β (SE)**	**P_t**	**P_i**
rs3923113	GRB14	0.008 (0.01)	0.49	-0.003 (0.01)	0.81	0.004 (0.02)	0.79	0.80
rs1801282	PPAR-γ	0.02 (0.02)	0.29	-0.004 (0.02)	0.81	0.02 (0.02)	0.48	0.52
rs6878122	ZBED3	0.02 (0.01)	0.07	(0.01) (0.01)	0.20	0.02 (0.01)	0.13	0.28
rs4402960	IGF2BP2	-0.008 (0.01)	0.47	-0.004 (0.01)	0.75	-0.02 (0.01)	0.12	0.45
rs5945326	DUSP9	0.0008 (0.01)	0.93	0.02 (0.01)	0.10	0.03 (0.01)	0.03	0.04
rs849135	JAZF1	0.01 (0.01)	0.26	0.01 (0.01)	0.27	0.03 (0.01)	0.01	0.10
rs17791513	TLE4	-0.002 (0.01)	0.90	-0.009 (0.01)	0.52	0.02 (0.02)	0.29	0.45
rs11257655	CDC123	-0.02 (0.01)	0.11	0.006 (0.01)	0.68	-0.01 (0.02)	0.51	0.20
rs10830963	MTNR1B	0.005 (0.01)	0.67	0.01 (0.01)	0.43	0.013 (0.01)	0.47	0.83
rs7403531	RASGRP1	0.005 (0.01)	0.62	0.005 (0.01)	0.68	0.009 (0.01)	0.54	0.93
rs11717195	ADCY5	0.0002 (0.01)	0.98	-0.01 (0.01)	0.26	-0.01 (0.02)	0.42	0.53
rs7756992	CDKAL1	-0.02 (0.01)	0.02	-0.02 (0.01)	0.056	-0.02 (0.01)	0.12	0.12
rs516946	ANK1	0.02 (0.01)	0.10	0.009 (0.01)	0.54	0.03 (0.02)	0.10	0.25
rs2796441	TLE1	-0.02 (0.01)	0.10	-0.01 (0.01)	0.24	-0.02 (0.01)	0.18	0.37
rs7903146	TCF7L2	0.0004 (0.01)	0.97	0.020 (0.01)	0.11	0.05 (0.01)	**0.001**	**0.002**
rs7202877	BCAR1	0.001 (0.01)	0.94	-0.01 (0.01)	0.36	0.04 (0.02)	0.04	0.050

Log-glycemia was regressed SSB, additive SNP effects, and the interaction between these variables. The SNP genotype was coded as the number of D2M risk alleles in each person. The interaction βs represent de difference in betas for a SNP between SSB Category 1 and another SSB category. Results are expressed in β(Standard Deviation), 95% Confidence Interval (95%CI) and p-value. Models adjusted for: age, sex, BMI, waist circumference, physical activity, schooling, socioeconomic level, smoking, consumptions: fruit, vegetables, sugar, processed meat, alcoholic beverages and Amerindian ancestry. P values in bold are significant after Bonferroni correction ($p<0.0031$), P_t= P for trend and P_i= P for interaction, n=2828.

Supplementary Table S8. Interaction between SSB intake categories and genotypes of selected variants on fasting glucose (log).

			SSB Category 2		SSB Category 3		SSB Category 4		
**rsID**	**Gene**	**GT**	**β (SE)**	**P_t**	**β (SE)**	**P_t**	**β (SE)**	**P_t**	**P_i/R^2**
rs3923113	GRB14	C/C	4.26 (0.06)	0.20	4.38 (0.07)	0.49	4.35 (0.08)	0.85	0.97/
		C/A	0.02 (0.03)	0.52	0.003 (0.04)	0.93	0.01 (0.04)	0.80	0.0007
		A/A	0.03 (0.03)	0.42	-0.002 (0.04)	0.96	0.01 (0.04)	0.75	
rs1801282	PPAR-*γ*	G/G	4.39 (0.05)	0.48	4.40 (0.06)	0.42	4.29 (0.07)	0.76	0.87/
		G/C	-0.001 (0.07)	0.98	-0.03 (0.08)	0.68	0.03 (0.10)	0.80	0.0002
		C/C	0.02 (0.07)	0.78	-0.03 (0.07)	0.66	0.04 (0.10)	0.68	
rs6878122	ZBED3	A/A	4.26 (0.05)	0.27	4.3 (0.06)	0.63	4.24 (0.07)	0.73	0.27/
		A/G	0.016 (0.02)	0.32	0.02 (0.018)	0.18	0.04 (0.02)	0.038	0.005
		G/G	0.04 (0.02)	0.07	0.02 (0.03)	0.35	0.02 (0.03)	0.43	
rs4402960	IGF2BP2	G/G	4.23 (0.05)	0.37	4.31 (0.06)	0.52	4.28 (0.07)	0.92	0.15/
		G/T	0.006 (0.01)	0.68	0.020 (0.02)	0.22	0.005 (0.02)	0.80	0.01
		T/T	0.04 (0.028)	0.13	-0.04 (0.03)	0.15	-0.09 (0.04)	0.01	
rs5945326	DUSP9	G/G	4.26 (0.05)	0.23	4.326 (0.06)	0.92	4.3 (0.07)	0.80	0.07/
		G/A	0.008 (0.02)	0.64	0.04 (0.02)	0.05	0.003 (0.02)	0.90	0.007
		A/A	-0.002 (0.02)	0.92	0.03 (0.02)	0.22	0.05 (0.02)	0.030	
rs849135	JAZF1	A/A	4.23 (0.05)	0.12	4.34 (0.06)	0.79	4.26 (0.07)	0.45	0.17/
		A/G	0.04 (0.02)	0.09	0.01 (0.03)	0.62	0.06 (0.03)	0.04	0.004
		G/G	0.04 (0.02)	0.12	0.02 (0.03)	0.35	0.08 (0.03)	0.007	
rs17791513	TLE4	G/G	4.12 (0.07)	0.42	4.25 (0.07)	0.27	4.18 (0.01)	0.95	0.77/
		G/A	0.003 (0.05)	0.95	-0.03 (0.05)	0.48	-0.02 (0.09)	0.79	0.003
		A/A	0.001 (0.04)	0.97	-0.03 (0.04)	0.40	0.003 (0.09)	0.97	
rs11257655	CDC123	C/C	4.23 (0.05)	0.39	4.31 (0.06)	0.44	4.27 (0.07)	0.97	0.45/
		C/T	-0.01 (0.01)	0.34	0.002 (0.02)	0.92	-0.017 (0.02)	0.41	0.004
		T/T	-0.04 (0.03)	0.18	0.03 (0.04)	0.39	0.003 (0.04)	0.94	
rs10830963	MTNR1B	C/C	4.21 (0.05)	0.26	4.30 (0.06)	0.61	4.28 (0.07)	0.88	**0.001/**
		C/G	0.01 (0.02)	0.54	0.014 (0.018)	0.41	-0.03 (0.02)	0.16	0.01
		G/G	-0.004 (0.04)	0.91	0.014 (0.05)	0.75	0.19 (0.05)	**0.0008**	
rs7403531	RASGRP1	C/C	4.24 (0.35)	0.35	4.34 (0.41)	0.41	4.27 (0.81)	0.81	0.67/
		C/T	-0.01 (0.01)	0.46	-0.01 (0.01)	0.87	-0.008 (0.02)	0.68	0.003
		T/T	0.04 (0.03)	0.13	0.02 (0.03)	0.45	0.04 (0.04)	0.24	
rs11717195	ADCY5	C/C	4.17 (0.06)	0.34	4.26 (0.06)	0.54	4.21 (0.09)	0.87	0.66/
		C/T	-0.005 (0.03)	0.84	0.009 (0.03)	0.78	0.019 (0.04)	0.66	0.004
		T/T	0.004 (0.03)	0.88	-0.015 (0.03)	0.62	-0.005 (0.04)	0.90	
rs7756992	CDKAL1	A/A	4.25 (0.05)	0.46	4.34 (0.06)	0.35	4.33 (0.07)	0.56	0.03/
		A/G	-0.02 (0.01)	0.22	-0.009 (0.017)	0.58	-0.05 (0.02)	0.01	0.006
		G/G	-0.05 (0.02)	0.02	-0.05 (0.02)	0.03	-0.02 (0.03)	0.31	
rs516946	ANK1	T/T	4.22 (0.07)	0.22	4.33 (0.07)	0.65	4.2 (0.09)	0.76	0.62/
		T/C	0.005 (0.04)	0.89	0.008 (0.05)	0.85	0.004 (0.06)	0.94	0.007
		C/C	0.03 (0.04)	0.44	0.02 (0.04)	0.69	0.04 (0.05)	0.45	
rs2796441	TLE1	A/A	4.22 (0.05)	0.34	4.31 (0.06)	0.47	4.27 (0.07)	0.96	0.59/
		A/G	-0.006 (0.02)	0.75	-0.0001 (0.02)	0.99	0.001 (0.02)	0.96	0.003
		G/G	-0.04 (0.02)	0.08	-0.03 (0.02)	0.23	-0.04 (0.03)	0.16	
rs7903146	TCF7L2	C/C	4.23 (0.05)	0.32	4.32 (0.06)	0.44	4.26 (0.07)	0.80	0.005/
		C/T	-0.006 (0.02)	0.72	0.03 (0.02)	0.10	0.03 (0.02)	0.09	0.009
		T/T	0.009 (0.03)	0.77	0.02 (0.03)	0.53	0.14 (0.04)	**0.0006**	
rs7202877	BCAR1	G/G	4.24 (0.05)	0.32	4.33 (0.06)	0.44	4.27 (0.07)	0.80	0.15/
		G/T	-0.02 (0.02)	0.73	-0.05 (0.02)	0.40	-0.04 (0.02)	0.55	0.005

T/T	-0.02 (0.03)	0.76	-0.06 (0.03)	0.28	0.004(0.04)	0.96

Log-glycemia was regressed SSB, SNP genotype, and the interaction between these variables. The SNP genotype was coded as the combination of alleles, using the nitrogenous base in the forward strand to denote each allele. The first genotypes in each SNP is the homocigotefor the protective allele, which was used as a baseline level. The βs for the first interaction effects, i.e. the change in log-glycemie due to the combination of genotype and consumption level. Models were adjusted for: age, sex, BMI, waist circumference, physical activity, schooling, socioeconomic level, smoking, consumptions: fruit, vegetables, sugar, processed meat, alcoholic beverages and Amerindian ancestry. P values in bold are significant after Bonferroni correction ($p<0.0031$), R^2: percentage of the variance explained by each SNP by $R^2=SS_{SNP}/SS_{Tot}$, were SS_{SNP} is the sum of squares due to the SNP's marginal and interaction effects and SS_{SNP} is the total sum of squares, $P_{t=}$ P for trend and $P_{i=}$ P for interaction, n=2828.

Supplementary Table S9. Association between SNPs (categorical) and fasting glucose (log) (continuous) and Pos-Hoc power estimation in 2828 subjects.

rsID	Gen	GT	β (SE)	*P*	% Allelle Frequency MAUCO (n)	Pos-Hoc Power estimation	OR estimation
rs3923113	GRB14	C/C	Ref	0.99	5.8 (168)	-	
		C/A	0.0001 (0.01)		35.2 (1016)	3.2	1.00
		A/A	0.00006 (0.01)		59.0 (1699)	4.1	1.00
rs1801282	PPAR-γ	G/G	Ref	0.86	1.2 (33)	-	
		G/C	-0.009 (0.03)		17.6 (508)	5.9	0.99
		C/C	-0.006 (0.02)		81.2 (2340)	5.8	0.99
rs6878122	ZBED3	A/A	Ref	0.15	42.9 (1230)	-	
		A/G	0.003 (0.006)		44.7 (1280)	6.0	1.00
		G/G	0.02 (0.009)		12.4 (355)	34.1	1.02
rs4402960	IGF2BP2	G/G	Ref	**0.003**	50.6 (1442)	-	
		G/T	0.02 (0.006)		40.5 (1154)	86.8	1.02
		T/T	0.02 (0.01)		8.9 (252)	37.9	1.02
rs5945326	DUSP9	G/G	Ref	0.17	47.2 (1344)	-	
		G/A	-0.003 (0.007)		29.9 (850)	54,1	0.97
		A/A	0.01 (0.007)		22.8 (651)	67.8	1.01
rs849135	JAZF1	A/A	Ref	0.86	14.3 (411)	-	
		A/G	-0.0006 (0.008)		45.3 (1304)	5.7	0.99
		G/G	0.002 (0.008)		40.4 (1162)	3.3	1.00
rs17791513	TLE4	G/G	Ref	0.13	2.8 (81)	-	
		G/A	0.02 (0.02)		25.8 (746)	54.1	1.02
		A/A	0.03 (0.02)		71.3 (2060)	67.8	1.02
rs11257655	CDC123	C/C	Ref	0.07	61.0 (1746)	-	
		C/T	0.0005 (0.006)		33.8 (968)	3.4	1.00
		T/T	0.03 (0.01)		5.2 (149)	41.8	1.03
rs10830963	MTNR1B	C/C	Ref	0.18	64.4 (1837)	-	
		C/G	0.008 (0.006)		32.0 (914)	23.9	1.00
		G/G	0.02 (0.01)		3.6 (102)	21.4	1.02
rs7403531	RASGRP1	C/C	Ref	0.53	50.8 (1461)	-	
		C/T	-0.001 (0.006)		41.6 (1194)	5.0	0.99
		T/T	0.01 (0.01)		7.6 (219)	13.8	
rs11717195	ADCY5	C/C	Ref		6.9 (199)	-	
		C/T	-0.009 (0.01)	0.54	38.2 (1102)	19.8	0.99
		T/T	-0.004 (0.01)		54.9 (1581)	11.8	0.99
rs7756992	CDKAL1	A/A	Ref	0.35	42.7 (1229)	-	
		A/G	-0.006 (0.005)		44.3 (1276)	5.4	0.99
		G/G	-0.01 (0.008)		13.0 (375)	17.0	0.99
rs516946	ANK1	T/T	Ref	**0.0001**	3.1 (88)	-	
		T/C	0.02 (0.03)		28.4 (809)	48.5	1.02
		C/C	0.01 (0.004)		68.4 (1950)	91.2	1.01
rs2796441	TLE1	A/A	Ref	0.59	27.1 (780)	-	
		A/G	0.005 (0.006)		49.4 (1422)	27.9	1.00
		G/G	0.007 (0.007)		23.5 (677)	77.5	1.00
rs7903146	TCF7L2	C/C	Ref	0.43	57.8 (1639)	-	
		C/T	0.004 (0.006)		36.2 (1025)	7.1	1.00
		T/T	0.01 (0.01)		6.0 (169)	8.7	1.01
rs7202877	BCAR1	G/G	Ref	0.43	1.9 (54)	-	
		G/T	0.01 (0.02)		21.1 (606)	12.6	1.01

	T/T	0.009 (0.02)		77.0 (2213)	5.3	1.00

The reference corresponds to the coefficient of the protection homozygote (0 risk alleles), SE: Standard Error, P_t: p value for trend, P_i: P value for interaction. Model adjusted for: age, sex, BMI, waist circumference, physical activity, education, socio-economic level, smoking, consumptions: fruit, vegetables, sugar, processed meats, alcoholic beverages and % Amerindian ancestry. P_{t}= P for trend and P_{i}= P for interaction. P values in bold are significant after Bonferroni correction (p<0.0031). Pos-hoc power was estimated using the online web tool (https://clincalc.com/stats/power.aspx). OR was estimated using e^beta. P values in bold are significant after Bonferroni correction (p<0.0031).

Supplementary Table S10. Sensitivity Analysis. Interaction between SSB categories and rs7903146 and rs10830963 loci (as continuous and categorical variables) in subjects younger than 65 years.

**rsID**	**Gen**	**Genotype**	**SSB Category 2**		**SSB Category 3**		**SSB Category 4**		
			**β (SE)**	**P_t**	**β (SE)**	**P_t**	**β (SE)**	**P_t**	**P_i**
rs7903146	TCF7L2	0,1,2	-0.003 (0.01)	*0.81*	0.02 (0.01)	*0.25*	0.04 (0.02)	*0.07*	0.01
		C/C	Ref		Ref		Ref		
		C/T	-0.004 (0.02)	0.79	0.007 (0.03)	0.83	0.03 (0.02)	0.21	0.03
		T/T	0.01 (0.04)	0.70	0.02 (0.2)	0.28	0.13 (0.04)	0.003	
rs10830963	MTNR1B	0,1,2	0.0055 (0.01)	0.71	0.01 (0.02)	0.40	-0.03 (0.02)	0.13	0.14
		C/C	Ref		Ref		Ref		
		C/G	0.02 (0.01)	0.37	0.02 (0.02)	0.92	-0.04 (0.02)	0.10	0.20
		G/G	-0.02 (0.04)	0.71	0.01 (0.05)	0.84	-0.02 (0.06)	0.77	

The reference corresponds to the coefficient of the protection homozygote (0 risk alleles), SE: Standard Error, P_t: p value for trend, P_i: p value for interaction. Model adjusted for: age, sex, BMI, waist circumference, physical activity, education, socio-economic level, smoking, consumptions: fruit, vegetables, sugar, processed meats, alcoholic beverages and % Amerindian ancestry. P_t= P for trend and P_i= P for interaction. P values in bold are significant after Bonferroni correction (p<0.0031), n=2454.

Supplementary Table S11. Sensitivity Analysis. Interaction effects between GRSw and sugar-sweetened beverages intake on fasting blood in subjects less than 65 years old.

SSB	β (SE)	P_t	P_i
SSB and GRSw as continuous			
	0.07 (0.006)	**0.02**	**0.04**
Categorical SSB and continuous GRSw			
2	0.0002 (0.02)	0.98	0.15
3	0.01 (0.02)	0.57	
4	0.05 (0.02)	**0.04**	
Categorical SSB/ GRSw tertile 2			
2	0.03 (0.02)	0.17	0.14
3	0.03 (0.02)	0.14	
4	-0.006 (0.02)	0.81	
Categorical SSB/ GRSw tertile 3			
2	0.0009 (0.02)	0.96	
3	0.02 (0.02)	0.39	
4	0.03 (0.02)	0.20	

β estimated interaction effect, SE: Standard Error, Pt: P value for trend, Pi: P value for interaction. Models adjusted for: age, sex, BMI, waist circumference, physical activity, schooling, socio-economic level, smoking, consumptions of fruit, vegetables, sugar, processed meat, and alcoholic beverages, and Amerindian ancestry, P values in bold are significant ($p < 0.05$), n=2454.

Supplementary Table S12. Sensitivity Analysis. Interaction effect between SNPs (rs7903146 and rs10830963) and 2 levels of SSB consumption (continuous and categorical) and two levels of SSB consumption
on log-fasting glucose.

**rsID**	**Gen**	**Genotype**	**SSB Category 2**		
			**β (SE)**	**P_t**	***P_i***
rs7903146	TCF7L2	0,1,2	0.03 (0.01)	0.001	**0.001**
		C/C	Ref		
		C/T	0.03 (0.01)	0.007	**0.006**
		T/T	0.06 (0.03)	0.02	
rs10830963	MTNR1B	0,1,2	0.80 (0.01)	0.42	0.42
		C/C	Ref		
		C/G	-0.007 (0.01)	0.59	0.056
		G/G	0.07 (0.03)	0.02	

The reference corresponds to the coefficient of the protection homozygote (0 risk alleles), SE: Standard Error, P_t: p value for trend, P_i: p value for interaction. Model adjusted for: age, sex, BMI, waist circumference, physical activity, education, socio-economic level, smoking, consumptions: fruit, vegetables, sugar, processed meats, alcoholic beverages and % Amerindian ancestry. P values in bold are significant after Bonferroni correction (p<0.0031), n=2828.

Supplementary Table S13. Sensitivity Análisis. Interaction effect between GRSw (continous and categorical) and 2 levels of SSB consumption (continuous and categorical) on log-fasting blood glucose.

SSB	β (SE)	P_t	P_i
**SSB and GRSw as continuous**			
	0.01 (0.06)	0.04	**0.04**
**Categorical SSB and continuous GRSw**			
**2**	0.02 (0.01)	0.04	**0.04**
**Categorical SSB**	**GRSw tertile 2**		
**2**	0.0002 (0.01)	0.98	0.12
**Categorical SSB**	**GRSw tertile 3**		
**2**	0.03 (0.01)	0.06	0.07

β estimated interaction effect, SE: Standard Error, Pt: p value for trend, Pi: p value for interaction. Models adjusted for: age, sex, BMI, waist circumference, physical activity, schooling, socio-economic level, smoking, consumptions of fruit, vegetables, sugar, processed meat, and alcoholic beverages, and Amerindian ancestry, n=2828.
